# Selenium Nutritional Status Assessment in Chinese Adult Females: Results from the China Nutrition and Health Surveillance in 2015

**DOI:** 10.3390/nu17091427

**Published:** 2025-04-24

**Authors:** Jie Feng, Yang Cao, Huidi Zhang, Jingxin Yang, Wenxuan Wu, Jiaxi Lu, Lichen Yang

**Affiliations:** 1 Key Laboratory of Public Nutrition and Health, National Health Commission of the People’s Republic of China, Beijing 100050, China; 2 National Institute for Nutrition and Health, Chinese Center for Disease Control and Prevention, Beijing 100050, China; 3 School of Child Development and Education, China Women’s University, Beijing 100101, China

**Keywords:** plasma selenium, dietary selenium, Chinese adult females, cross-sectional study

## Abstract

Objectives: Although some studies have assessed Selenium nutritional status in different populations, determining the plasma Selenium levels and describing the distribution of dietary Selenium intake in Chinese female adults by using nationally representative data was lacking. The objective was to describe the plasma/dietary Selenium status in Chinese female adults and analyze the possible influencing factors related to dietary Selenium levels. Method: A total of 3016 female adults from China Nutrition and Health Surveillance in 2015 (CNHS 2015) were included. The plasma Selenium concentration was detected by inductively coupled plasma mass spectrometry (ICP-MS). Dietary Selenium intake (Y, μg/d) was calculated from plasma Selenium concentrations (X, μg/L) using the formula lg(Y) = 1.624 lg(X) + 3.389. A multivariate logistic regression model was used to explore the risk factors of low dietary Selenium intake. Results: The median levels of plasma Selenium and dietary Selenium in Chinese adult females were 89.97 μg/L and 49.03 μg/d, respectively. The normal reference range of plasma Selenium in this population was 72.04~141.11 μg/L. There was a higher risk of low dietary Selenium intake in central, western, and northern regions. In general, the plasma Selenium levels in Chinese adult females were lower than those in countries such as the United States and Japan but higher than those in some European countries. Conclusions: The plasma Selenium levels varied greatly in different regions of China, with typical regional characteristics. Therefore, it was necessary to monitor Selenium nutrition monitoring in specific regions.

## 1. Introduction

Selenium (Se) is an essential trace element for humans. It is a component of the amino acid selenocysteine present in the active site of the Se-dependent enzymes (e.g., glutathione peroxidases). Se exerts its biological function through selenoproteins. Most of the selenoproteins are involved in the regulation of antioxidant defense and redox state, and the glutathione peroxidase family is involved in the cellular oxidative stress defense system and in the maintenance of intracellular redox state to maintain the vitality of cells and body [1,2].

As a “dual-surface” element, Se has a narrow range between dietary deficiency and toxic concentration [3]. For example, the recommended nutrient intake (RNI) and the maximum tolerable intake (UL) of Se for Chinese adults are 60 μg/d and 400 μg/d, respectively, while the corresponding values for American adults are 55 μg/d and 400 μg/d, respectively [4,5]. Se deficiency will lead to typical local diseases, such as Kashin-Beck disease and Keshan disease [6]. It is worth noting that patients with Keshan disease are mostly seen in women and children [7]. Insufficient Se levels in women may have adverse effects on the health of the next generation [8]. Studies have shown that women with low Se levels have relatively low fertility compared with high Se levels [9]. At the same time, some meta-analyses of observational studies have suggested that low Se levels may be associated with breast cancer [10,11] and depression [12]. On the other hand, excessive Se may also cause “Se poisoning”. Numerous experimental and observational studies have suggested that Se exposure may increase the risk of type 2 diabetes across a wide range of exposure levels [13]. However, other studies have revealed nonlinear (U-shaped) associations or null associations [14,15]. Interestingly, several studies have found that females may face a higher risk of type 2 diabetes [16,17]. Therefore, it is of very important public health significance to assess the Se nutritional status of females reasonably and accurately and to take timely interventions to reduce the risk of related diseases.

There are many types of samples evaluating the Se nutritional status of the population, including diet, blood (whole blood, plasma, serum, blood cells), urine, etc. Plasma Se, although generally not considered to be an ideal biomarker of Se status, was the most widely used in most countries to evaluate the Se nutritional status of the population using nutrition surveillance data. For example, the serum/plasma Se levels in the United States and Japan populations were at a relatively high level (>120 μg/L) [18,19]. A French study that included 1821 adult females had a mean plasma Se level of 85.32 μg/L [20]. Although the Se nutritional status of populations in different regions of China has been reported, there was still a lack of assessment of the Se nutritional status of a nationally representative population.

Notably, China is one of the 40 countries designated as low-Se or Se-deficient according to the World Health Organization (WHO) [21], making the assessment of population Se status particularly relevant for nutritional guidance. Therefore, the present study aimed to evaluate the plasma Se concentrations and dietary Se intake levels in Chinese adult females using the data of China Nutrition and Health Surveillance in 2015 (CNHS 2015), which was the nationwide survey on nutrition in China. Furthermore, the prevalence of low dietary Se intake and the potential risk factors in this population were assessed to guide surveillance priorities and inform subsequent targeted investigations.

## 2. Materials and Methods

### 2.1. Participant Characteristics

All the samples were selected from CNHS 2015. CNHS 2015 is a cross-sectional survey of the civilian non-institutionalized population of China, conducted by the National Institute of Nutrition and Health and the National Center for Chronic and Non-Communicable Disease Control and Prevention, Chinese Center for Disease Control and Prevention (NINH and NCNCD, China CDC). People with serious physical and mental diseases were excluded from the survey.

The minimum sample size of this study was calculated by the formula below [22].

N = (p × (1 − p) × u^2^)/d^2^ × deff (1)

N, number of samples; p, deficiency rate; u, confidential level, 1.96; d, allowance error, 2.7%; deff, design effect, 1.5. The reported serum/plasma Se deficiency rate of Chinese women of childbearing age was 24.04% [23]. The calculated minimum sample size was 2886. Based on the demographic information, all samples of adult females > 18 years in this study were selected from the biological samples’ bank established from CNHS 2015. Pregnant women were excluded. All the samples were selected by the method of simple random sampling. Considering the representativeness of urban and rural areas, the numbers of surveillance sites (a total of 289 sites), and the age distribution (18~29 y, 30~44 y, 45~55 y, 55~65 y, >65 y), a total of 3016 adult females were randomly selected in this study.

### 2.2. Basic Information and Evaluation Standards

Demographic characteristics information was collected with a standardized questionnaire, including age, nationality, education, residence region (urban and rural), location (eastern, central, and western), latitude, drinking, and smoking situation. Among them, height, weight, waist circumference, and blood pressure were measured by trained medical staff following standardized procedures. The body mass index (BMI) data were calculated by height and weight. The details of collection, storage, and clinical index detected of venous blood can be found in the study by Zhang et al. [24]. Participants were identified as a drinker or a smoker if they had a history of drinking or smoking, respectively.

### 2.3. Determination of Plasma Se Concentration

Plasma samples were diluted with 0.5% (*v*/*v*) HNO_3_ (1:20), and Se concentration in the plasma was measured by inductively coupled plasma mass spectrometry (ICP-MS, PerkinElmer, NexION 350, Waltham, MA, USA). More details on the method of Se concentration determination can be found in Cao et al. [23]. The rate of recovery of plasma Se was 113.14%. The coefficient of variation between and within batches of Se was 2.66% and 1.54%. The limit of detection (LOD) was 0.43 μg/L, and the limit of quantitation (LOQ) was 1.43 μg/L for plasma Se.

### 2.4. Calculation of Dietary Se Intake

The geographical environment of Se distributions is complex in China, which leads to big differences in food Se content from different areas. Dietary Se intake data were obtained by formula calculation from plasma Se level in this study. The formula was lgY = 1.624 lg(X) + 3.389 (X = plasma Se concentration in mg/L; Y = daily Se intake in μg/d), which was derived from the extensive studies of Yang et al. [25]. This formula was derived from the population data across regions with low, medium, and high Se levels in China. It has been formally adopted in the latest edition of the Chinese Dietary Reference Intakes (DRIs 2023).

### 2.5. Statistical Analysis

IBM SPSS version 26.0 software was used for data cleaning and analysis. Basic information and Se level were expressed as median (P_25_–P_75_) for skewed distribution. The differences between the subgroups were tested by the Kruskal–Wallis and Chi-square tests for categorical variables. The median of dietary Se intake of adult females was calculated by plasma Se data at each monitoring site. A multivariate logistic regression model was used to explore the risk factors of low dietary Se intake, which was described as the odds ratios (ORs) and 95% confidence intervals (95% CI). All statistical tests were two-sided, and statistical significance was considered at *p* < 0.05.

## 3. Results

### 3.1. Characteristics of the Participants

After excluding unqualified samples, including incomplete basic information and poor plasma sample quality, a total of 3016 adult female participants were included in this cross-sectional study. The median age of the participants was 39 (27~58) years old, with a height of 156.0 (151.6~160.0) cm, weight of 56.4 (50.0~63.4) kg, waist circumference of 78.03 (71.76~85.35) cm, and a constitutional index (BMI) of 23.23 (20.91~25.93) kg/m^2^.

The plasma Se concentrations and dietary Se intake levels of the participants are shown in Table 1. The median concentration of plasma Se in adult females was 89.97 μg/L. Plasma Se concentrations varied significantly in terms of ethnic groups, geographical location, latitude, and residence region (*p* < 0.05). No group differences were observed in age, physical index, or drinking and smoking variables (*p* > 0.05).

The median level of dietary Se intake calculated by the formula from plasma Se was 49.03 μg/d. In the comparison of different stratification factors, Han ethnic females had higher dietary Se intake than other ethnic minorities (*p* < 0.05). Females living in eastern China had higher Se intake levels than those in central and western China (*p* < 0.05); females living in southern China had higher Se intake levels than those in northern China (*p* < 0.05). Dietary Se intake in junior high school and above-level populations was higher than those in primary school and below, and the difference was statistically significant (*p* < 0.05). There was no statistical difference between the distribution levels of age, BMI, smoking, and alcohol consumption groups on dietary Se intake (*p* > 0.05).

### 3.2. Reference Interval of Plasma Se for Adult Females

To evaluate the nutritional status of Se in adult females, the reference interval of plasma Se levels should be determined in advance. To establish the reference interval of plasma Se, 316 healthy adult females were selected from 3016 adult females with strict inclusion and exclusion criteria described in our previous study [26]. The BMI, blood lipid, blood pressure, blood glucose, etc., of all the individuals were within the normal range. The basic demographic and clinical data were presented in Table 2. The reference interval of plasma Se levels in this healthy population was established based on the P_2.5_~P_97.5_ range of plasma Se concentration, which was 72.04~141.22 μg/L (see Table 3).

### 3.3. Comparison of Se Nutritional Status Among Different Groups in 3016 Adult Females

The distribution of plasma Se levels in 3016 adult females was analyzed according to the reference interval values established in this study, and the results were shown in Table 4. In Chinese adult females, 72.75% of the population had adequate plasma Se levels, 22.78% of those had plasma Se levels lower than 72.04 μg/L, and 4.48% of those had plasma Se levels higher than 141.22 μg/L. Among them, there were significant differences in the group distribution of age, nationality, geographical location, latitude, urban and rural types, and education level.

According to the Dietary Reference Intakes for China (2023 edition), the estimated average requirement (EAR) and recommended nutrient intake (RNI) of Se for the Chinese population are 50 and 60 μg/d, respectively; the maximum tolerable intake of Se is 400 μg/d [4]. The description of the prevalence of dietary Se intake distribution in this study was shown in Table 5. The overall prevalence of dietary Se intake levels lower than EAR, between EAR~RNI, and higher than RNI but below UL value was 52.59%, 16.25%, and 31.17%, respectively. No participants exceeded the UL for dietary Se intake.

### 3.4. The Risk of Low Dietary Se Intake with Multivariate Logistic Regression Analysis

We adopted the criteria for determining low dietary Se intake as recommended by the DRIs, with a cut-off value of <50 μg/d for adult females. The explorations of the risk factors associated with low dietary Se intake were shown in Table 6. Among the continuous variables, TG was found to be a risk factor for the low dietary Se intake, with the OR (95% CI) value being 1.17 (1.03–1.32, *p* < 0.05). Fasting blood glucose was found to be a protective factor for the low dietary Se intake, with the OR (95% CI) value being 0.87 (0.80–0.94, *p* < 0.05).

Among the classification variables, compared with the population in eastern China, the OR for low dietary Se intake was 1.60 (95% CI: 1.32–1.92, *p* < 0.05) in central China and 3.62 (95% CI: 2.95–4.43, *p* < 0.05) in western China. Compared with southern China, northern China had an OR of 2.41 (95% CI: 2.04–2.85, *p* < 0.05). No other continuous or categorical variables showed significant associations with low dietary Se intake in the analysis.

## 4. Discussion

In the present study, plasma Se concentrations and dietary Se intake levels were determined to evaluate the Se nutritional status of Chinese adult females based on the nationally representative population from CNHS 2015. The median plasma Se level of Chinese adult females was 89.97 μg/L, and a significant difference was observed between ethnicity, geographical location, latitude, and urban and rural types. Meanwhile, we also obtained the normal reference range of plasma Se for this population (72.04~141.22 μg/L).

The distribution and comparative studies of plasma/serum Se levels in different countries were shown in Table 7. Serum Se (mean ± SD) in American adults over 40 years of age was 127.5 ± 17.76 μg/L using NHANES data [19]. Plasma Se (mean ± SD) in Korean females aged 12~78 years was 110.06 ± 19.68 μg/L, with no association between Se level and age [27]. Se levels (mean ± SD) in healthy Swiss females were 95.6 ± 13.3 μg/L, and the effect of regional differences was also found [28]. The Se levels in the above countries were higher than those in the Chinese population. The serum Se concentrations in Ethiopian and Greek females were similar to those in Chinese females [29,30]. Females in the UK [31], Serbia [32], and Germany [33] had lower Se levels compared with the Chinese population. Generally low Se concentration in Europe may be due to the low Se concentration in the environmental soil, which made the Se intake generally low in the population, thus affecting the Se concentration in the body [34]. In general, the plasma Se levels in Chinese adult females were lower than those in countries such as the United States and Japan but higher than those in some European countries.

The plasma/serum Se levels varied greatly in different regions of China, with typical regional characteristics. The serum Se concentration of Shanghai people (103.29 μg/L) [35] was higher than the national average level. Levels in Linzhou (85.9 μg/L), Henan province [36], were close to our results, whereas those in Zhoukoudian (75.01 μg/L), Beijing [21], and Taiyuan (79.38 μg/L), Shanxi province [37], were lower than our findings. Environmental Se analysis in Zhoukoudian revealed that while topsoil Se levels fell within the adequate range, both water Se and grain Se concentrations were in the deficient-to-marginal range [21].

Plasma Se concentration in central and western China was low compared with this study, such as in Ningshan, Shaanxi province (58.0 μg/L) [38], and in Tibet (26.29 μg/L) [39] in China. The low serum Se level of the Tibetan population may be due to the diet heavily reliant on barley and tsampa (low in Se level), with minimal intake of Se-rich foods (such as eggs, beans, and meat) [40], leading to the poor nutritional status of the local population.

In the previous reports, dietary Se intake was calculated using dietary surveys (24 h review or FFQ method) combined with Food Composition Tables [41,42,43,44]. The geographical environment of Se distributions is complex in China. Se concentrations in soils, plants, and animals are fundamentally determined by geology [45]. Nevertheless, there was no data specific to food Se content for those in high-Se or low-Se geographical regions [46]. The Food Composition Table cannot adequately reflect the influence of geographical differences on Se concentration in food. Based on the Nutrition and Health Monitoring Report of the Chinese Population (2015–2017), which also used the CACDNS (2015) database, calculated dietary Se intake by Food Composition Table for 18~59-year-old females was 36.9 μg/d, and for females over 60 years old, it was 32.5 μg/d [47]. In our present study, dietary Se data (Y, μg/day) were calculated from plasma Se concentration (X, mg/L) using the formula lgY = 1.624 lg(X) + 3.389. The formula for converting plasma Se to dietary Se intake in this study was derived from extensive data across regions with low, medium, and high Se levels in Chinese populations, making it more suitable for our study. It has been officially adopted in the latest edition of the Chinese Dietary Reference Intakes (DRIs 2023). The median dietary Se intake level of Chinese adult females was 49.03 μg/d (P_25_–P_75_: 35.57~64.68 μg/d), which was close to the EAR level of 50 μg/d for the Chinese population. The dietary Se data calculated from the formula was higher than that from the Nutrition and Health Monitoring Report of the Chinese Population (2015–2017). As a result, the method of formula calculation avoided the variation of Se content in food from different regions. Similarly, daily Se intakes in lactating Chinese females [46] and school-age children from rural areas of China [48] were also calculated separately by using this formula. There were also foreign reports of the formula for calculating dietary Se by plasma Se [49].

Plasma Se concentrations and dietary Se intake levels varied among subgroups, such as nationality, location, latitude, and residence region, in our present research. In a previous study, Galan et al. [20] also found that Se level in the French population was affected by geographical location and education level. Moreover, Burri et al. [28] found that serum Se concentration was affected by regional differences. The Se content in foods varies between regions or latitudes, leading to large differences in dietary Se concentrations in different regions. In the western part of China, the low dietary Se intake may be related to unbalanced diet patterns [39]. Xu et al. [50] also reported that the dietary Se levels of participants in rural areas were lower than those of participants living in urban areas, which was the same as the result of this study. The reasons for the differences in the dietary composition and structure of residents in different regions were related to differences in their income levels and patterns of expenditure.

In the exploration of the risk factors of low dietary Se intake in this study, it was found that fasting blood glucose was a protective factor, with a unit of increase in blood glucose associated with a 0.13 lower risk of low dietary Se intake. It is well known that the relationship between Se and type 2 diabetes mellitus (T2DM) is highly complex. Many researchers have investigated the effects of Se levels on T2DM, but the conclusions are controversial. A total of five studies revealed a U-shaped non-linear dose-response relation between serum Se and T2DM [15]. Results of several recent epidemiologic studies consistently indicated a positive association between dietary Se intake and the risk of T2DM [13,43], while others indicated a negative correlation or no significant association [51,52,53]. Furthermore, the Se status and intake varied widely in different parts of the world, which might cause the inconsistencies observed in study outcomes. Therefore, it was of great significance for a more in-depth exploration of the dose-response relationship between Se exposure and abnormal blood glucose for Chinese adult females.

Additionally, the rural city-type was found to increase the risk of low Se dietary intake. This may be caused by different economic levels and eating habits between urban and rural areas. In addition, people in western and northern China were more prone to have low Se dietary intake. Geographic location was a typical factor affecting Se intake in China. Se concentrations in soil, plants, and animals are essentially determined geologically. There is a “low selenium geological zone” from northeast to southwest, and the soil Se concentration in this area is lower compared with other areas [45], which may lead to low dietary Se levels.

There were some strengths in this study. To our knowledge, it was the first to evaluate and analyze the Se nutrition status of Chinese adult females based on a nationally representative surveillance. Here, the dietary Se intake levels were calculated from plasma Se concentration to avoid the bias of dietary Se intake caused by the variation of Se content in the same food in different regions. Most importantly, the reference range of plasma Se distribution of healthy Chinese adult females was established. Therefore, the cutoff values could be adopted to evaluate the Se nutrition status in this population. In spite of this, the limitation of this study was its cross-sectional design, which could only observe the association but not the causal relationship between the variables. Therefore, longitudinal studies and randomized controlled trials are needed to better elucidate the link.

## 5. Conclusions

In conclusion, relying on the biological samples and database of CNHS 2015, the plasma Se of Chinese adult females and the normal reference range of plasma Se in this population were obtained. The dietary Se levels of the population were described by the formula from the plasma Se level. The median dietary Se intake in Chinese adult females was 49.03 μg/d, which was close to the EAR (50 μg/d). The proportion of adult females with dietary Se intake below EAR was 52.29%. Besides, our study showed that there was a higher risk of low dietary Se intake in central, western, and northern regions. Therefore, the long-term monitoring and improvement of Se nutritional status should be strengthened for specific populations in this region.

## Figures and Tables

**Table 1:** Plasma Se and dietary Se intake concentrations of 3016 adult females in relation to different variables.

Variables	N	Plasma Se		Dietary Se Intake	
		Median (P_25_–P_75_) (μg/L)	*p*	Median (P_25_–P_75_) (μg/d)	*p*
**Total**	3016	89.97 (73.84–106.71)		49.03 (35.58–64.68)	
**Age (years)**					
18~44	1910	89.68 (74.55–105.11)	0.413	48.77 (36.13–63.11)	0.413
45~64	647	90.54 (74.49–108.61)		49.53 (36.08–66.56)	
≥65	459	90.65 (70.04–111.61)		49.11 (32.65–69.58)	
**BMI (kg/m^2^)**					
<18.5	192	89.86 (67.59–103.00)	0.630	48.93 (30.81–61.07)	0.63
18.5~23.9	1573	90.03 (74.29–106.47)		49.08 (35.92–64.45)	
24~27.9	849	90.12 (73.68–107.52)		49.17 (35.44–65.48)	
>28	402	89.24 (73.92–109.02)		48.39 (35.64–66.98)	
**Nationality**					
Han	2649	90.40 (75.25–106.91)	<0.05	49.41 (36.68–64.88)	<0.05
Ethnic minorities	367	83.66 (65.82–105.34)		43.57 (29.51–63.34)	
**District**					
Eastern	1010	96.92 (83.35–111.61)	<0.05	55.33 (43.31–69.58)	<0.05
Central	1016	90.79 (77.46–105.52) ^a^		49.76 (38.45–63.52) ^a^	
Western	990	79.65 (62.52–97.52) ^a,b^		40.23 (27.15–55.89) ^a,b^	
**Latitude, °** **N**					
≤33	1593	94.87 (79.50–111.56)	<0.05	53.44 (40.11–69.52)	<0.05
>33	1423	85.08 (68.83–100.72)		44.78 (31.73–58.89)	
**Residence region**					
Urban	1812	90.90 (77.11–106.52)	<0.05	49.85 (38.16–64.50)	<0.05
Rural	1204	87.42 (69.45–106.96)		46.80 (32.20–64.93)	
**Education**					
Primary school and blow	1268	88.42 (71.45–108.23)	0.053	47.66 (33.72–66.19)	0.053
Junior High School and Above	1748	90.53 (75.80–105.94)		49.52 (37.12–63.93)	
**Drinking**					
Yes	563	90.06 (73.53–106.91)	0.983	49.11 (35.33–64.88)	0.983
No	2453	89.05 (75.31–106.01)		48.22 (36.73–64.00)	
**Smoking**					
Yes	83	83.76 (68.48–101.18)	0.131	43.65 (31.48–59.33)	0.131
No	2933	90.06 (73.96–106.79)		49.11 (35.67–64.76)	

^a^: *p* < 0.05 compared with the population living in the eastern region; ^b^: *p* < 0.05 compared with the central region.

**Table 2:** General characteristics of the 316 healthy adult females.

Variables	Mean	Standard Deviation	Median (P_25_–P_75_)
Age (years)	39.9	14.7	37.3 (25.9–51.3)
Height (cm)	156.2	6.2	156.0 (152.3–160.0)
Weight (kg)	51.8	5.5	51.6 (48.1–55.5)
Waist (cm)	72.4	5.5	72.3 (68.5–77.0)
BMI (kg/m^2^)	21.2	1.4	21.2 (20.1–22.3)
FG (mmol/L)	4.92	0.39	4.92 (4.65–5.20)
HbA1c (%)	4.79	0.42	4.80 (4.43–5.10)
SBP (mmHg)	117.42	10.21	117.00 (109.67–124.67)
DBP (mmHg)	71.79	6.65	71.00 (66.67–76.67)
TC (mmol/L)	4.21	0.50	4.25 (3.85–4.61)
TG (mmol/L)	0.83	0.29	0.77 (0.61–1.02)
LDL-C (mmol/L)	2.37	0.43	2.43 (2.06–2.73)
HDL-C (mmol/L)	1.44	0.23	1.40 (1.26–1.59)
Hb (g/L)	137.28	15.02	138.82 (130.49–145.75)
UA (μmol/L)	241.58	52.22	241.55 (202.78–278.95)

Note: BMI—body mass index; FG—fasting glucose; HbA1c—hemoglobin A1c; SBP—systolic blood pressure; DBP—diastolic blood pressure; TC—total cholesterol; TG—triglyceride; LDL—low-density lipoprotein; HDL—high-density lipoprotein; Hb—hemoglobin; UA—uric acid.

**Table 3:** Plasma Se concentrations in 316 healthy adult females.

Variables	N	Plasma Se (μg/L)			
		Mean	Median	P_2.5_	P_97.5_
Total	316	100.43	99.52	72.04	141.22

**Table 4:** Comparison of plasma Se levels among different groups in 3016 subjects (%, 95% CI).

Characteristics	<72.04 μg/L		72.04~141.22 μg/L		>141.22 μg/L		*p*
	%	95% CI	%	95% CI	%	95% CI	
**Total**	22.78	21.23–24.24	72.75	71.15–74.40	4.48	3.75–5.21	
**Age (years)**							
18~44	22.20	20.31–24.11	73.72	71.74–75.69	4.08	3.20–4.97	<0.05
45~64	21.17	18.02–24.32	74.81	71.46–78.15	4.02	2.50–5.53	
≥65	27.45	23.37–31.54	65.80	61.45–70.14	6.75	4.46–9.05	
**BMI (kg/m^2^)**							
<18.5	29.69	23.44–36.46	66.15	59.38–72.92	4.17	1.56–7.29	0.310
18.5~23.9	21.55	19.52–23.59	73.81	71.67–75.97	4.64	3.62–5.72	
24~27.9	23.09	20.38–25.91	72.79	69.73–75.69	4.12	2.83–5.54	
>28	23.63	19.51–27.86	71.64	67.16–75.87	4.73	2.74–6.97	
**Nationality**							
Han	21.33	19.78–22.99	74.18	72.48–22.99	4.49	3.74–5.29	<0.05
Ethnic minorities	33.24	28.34–38.15	62.40	57.49–67.30	4.36	2.45–6.54	
**Location**							
Eastern	11.78	9.80–13.86	82.38	80.00–84.75	5.84	4.36–7.33	<0.05
Central	19.00	16.63–21.36	76.48	73.86–79.04	4.53	3.35–5.91	
Western	37.88	34.79–40.91	59.09	55.96–62.12	3.03	2.02–4.14	
**Latitude, °** **N**							
≤33	16.83	14.94–18.71	77.40	75.33–79.47	5.78	4.58–6.97	<0.05
>33	29.44	27.13–31.83	67.53	65.14–69.99	3.02	2.18–3.98	
**Residence region**							
Urban	19.26	17.49–21.14	76.99	75.00–78.92	3.75	2.87–4.64	<0.05
Rural	28.07	25.58–30.56	66.36	63.70–69.10	5.56	4.27–6.89	
**Education**							
Primary school and blow	25.95	23.58–28.39	69.01	66.40–71.53	5.05	3.86–6.31	<0.001
Junior High School and Above	20.48	18.71–22.31	75.46	73.40–77.45	4.06	3.15–4.98	
**Drinking**							
Yes	23.11	21.46–24.75	72.12	70.40–73.87	4.77	3.95–5.61	0.142
No	21.31	17.94–24.69	75.49	71.94–79.04	3.20	1.78–4.80	
**Smoking**							
Yes	22.60	21.07–24.14	72.96	71.36–74.57	4.43	3.68–5.18	0.228
No	28.92	19.28–38.55	65.06	55.42–74.70	6.02	1.20–12.05	

**Table 5:** Comparison of dietary Se intake levels among different groups in 3016 subjects (%, 95% CI).

Characteristics	<EAR (<50 μg/d)		EAR~RNI (50~60 μg/d)		>RNI (>60 μg/d)		*p*
	%	95% CI	%	95% CI	%	95% CI	
**Total**	52.59	50.76–54.34	16.25	14.99–17.64	31.17	29.51–32.76	
**Age (years)**							
18~44	53.30	51.06–55.54	17.49	15.78–19.19	29.21	27.17–31.26	0.014
45~64	51.16	47.31–55.01	14.84	12.10–17.58	34.00	30.35–37.66	
≥65	51.63	47.06–56.21	13.07	9.99–16.16	35.29	30.92–39.67	
**BMI (kg/m^2^)**							
<18.5	51.56	44.79–58.85	20.83	15.63–26.56	27.60	21.35–33.85	0.530
18.5~23.9	52.45	50.10–54.67	16.59	14.81–18.50	30.96	28.67–33.12	
24~27.9	52.65	49.23–56.07	15.55	13.31–18.02	31.80	28.74–34.98	
>28	53.48	48.51–58.21	14.18	10.95–17.91	32.34	27.62–36.57	
**Nationality**							
Han	51.30	49.38–53.26	17.18	15.78–18.68	31.52	29.67–33.26	<0.05
Ethnic minorities	61.85	56.40–66.49	9.54	7.08–12.81	28.61	24.25–33.51	
**Location**							
Eastern	39.41	36.34–42.18	18.81	16.53–21.19	41.78	38.81–44.95	<0.05
Central	50.79	47.93–53.94	18.90	16.54–21.26	30.31	27.46–33.07	
Western	67.88	64.95–70.91	10.91	8.99–12.83	21.21	18.69–23.84	
**Latitude, °** **N**							
≤33	43.44	40.99–45.83	18.33	16.45–20.21	38.23	35.91–40.74	<0.05
>33	62.83	60.30–65.43	13.91	12.16–15.74	23.26	21.01–25.51	
**Residence region**							
Urban	50.44	48.07–52.70	17.77	16.00–19.95	31.79	29.64–34.00	<0.05
Rural	55.81	53.08–58.55	13.95	12.04–15.95	30.23	27.57–33.05	
**Education**							0.040
Primary school and blow	54.34	51.58–57.02	14.35	12.46–16.48	31.31	28.78–33.99	
Junior High School and Above	51.32	48.97–53.55	17.62	15.96–19.51	31.06	29.00–33.27	
**Drinking**							
Yes	52.47	50.43–54.54	16.51	15.08–18.02	31.02	29.19–33.24	0.047
No	53.11	49.20–57.19	15.10	12.26–18.12	31.79	27.89–35.70	
**Smoking**							
Yes	61.45	50.60–72.29	14.46	7.23–22.89	24.10	15.66–33.73	0.245
No	52.34	50.63–54.28	16.30	15.00–17.76	31.37	29.56–33.00	

**Table 6:** Multivariate logistic regression model for risk factors associated with low dietary Se intake.

Variables		OR (95% CI)
Age (years)		1.00 (0.99–1.01)
BMI (kg/m^2^)		1.03 (0.99–1.06)
Waist (cm)		0.99 (0.98–1.00)
Hb (g/L)		1.00 (0.99–1.00)
UA (mmol/L)		1.00 (0.99–1.00)
Glucose parameters	FG (mmol/L)	0.87 (0.80–0.94) *
	HbA1c (%)	1.03 (0.91–1.17)
Blood Pressure (mmHg)	SBP	1.00 (0.996–1.01)
	DBP	1.00 (0.99–1.01)
Lipid (mmol/L)	TC	0.72 (0.47–1.10)
	TG	1.17 (1.03–1.32) *
	LDL-C	1.05 (0.69–1.62)
	HDL-C	0.98 (0.60–1.60)
Nationality	Han	1 (Ref)
	Ethnic minorities	1.08 (0.84–1.39)
Location	Eastern	1 (Ref)
	Central	1.60 (1.32–1.92) *
	Western	3.62 (2.95–4.43) *
Latitude, °N	≤33	1 (Ref)
	>33	2.41 (2.04–2.85) *
City-type	City	1 (Ref)
	Rural	1.19 (1.01–1.40) *
Education	Primary school and blow	1 (Ref)
	Junior High School and Above	0.87 (0.72–1.05)
Drinking	No	1 (Ref)
	Yes	1.15 (0.94–1.41)
Smoking	No	1 (Ref)
	Yes	1.37 (0.84–2.23)

Note: Hb—hemoglobin; UA—uric acid; * *p*-value < 0.05.

**Table 7:** Comparison of blood Se status in different countries and regions.

Years	Regions	Age (Years)	Gender	Sample Size	Blood State	Mean/Median (μg/L)	Method
	China (this study)	≥18	Female	3016	Plasma	89.97	ICP-MS
2020	USA [19]	≥40	Adult	1159	Serum	127.97	ICP-MS
2017	Korean [27]	12–78	Female	139	Serum	110.06	ICP-MS
2008	Swiss [28]	18–68	Female	656	Serum	95.6	ICP-MS
2020	Ethiopia [29]	15–49	Female	1327	Serum	94.8	ICP-MS
2009	Greece [30]	18–75	Female	210	Serum	93.9	ICP-MS
2013	UK [31]	19–64	Female	538	Plasma	84.53	ICP-MS
2020	Serbia [32]	18–40	Female	149	Serum	84.10	ICP-MS
2012	Germany [33]	≥20	Female	792	Serum	74.8	ICP-MS
2021	Shanghai, China [35]	≥35	Adult	1814	Serum	103.29	ICP-MS
2004	Henan, China [36]	≥40	Adult	205	Plasma	85.9	FS
2017	Shanxi, China [37]	≥18	Adult	1470	Serum	79.38	AAS
2007	Beijing, China [21]	15–84	Adult	401	Serum	75.01	GC
2020	Shaanxi, China [38]	18–70	Female	2889	Serum	58	AFS
2013	Xizang, China [39]	≥0	Adult	580	Serum	26.29	AAS

Note: ICP-MS: Inductively Coupled Plasma Mass Spectrometry; FS: Fluorescence Spectrometry; AAS: Atomic Absorption Spectrometry; GC: Gas Chromatograph; AFS: Atomic Fluorescence Spectrometry.

## Data Availability

The original contributions presented in the study are included in the article, further inquiries can be directed to the corresponding author.
